# *Trichoderma reesei* xylanase 5 is defective in the reference strain QM6a but functional alleles are present in other wild-type strains

**DOI:** 10.1007/s00253-017-8161-4

**Published:** 2017-02-22

**Authors:** Jonas Ramoni, Martina Marchetti-Deschmann, Verena Seidl-Seiboth, Bernhard Seiboth

**Affiliations:** 1 Molecular Biotechnology, Research Division Biochemical Technology, Institute of Chemical Engineering, TU Wien, Gumpendorferstraße 1a, 1060 Vienna, Austria; 2 Institute of Chemical Technologies and Analytics, TU Wien, 1060 Vienna, Austria

**Keywords:** XYN1, *Trichoderma reesei*, Xylanase, GH11 family, Recombinant protein production, Biorefinery, Biofuels

## Abstract

*Trichoderma reesei* is a paradigm for the regulation and industrial production of plant cell wall-degrading enzymes. Among these, five xylanases, including the glycoside hydrolase (GH) family 11 XYN1 and XYN2, the GH10 XYN3, and the GH30 XYN4 and XYN6, were described. By genome mining and transcriptome analysis, a further putative xylanase, encoded by *xyn5*, was identified. Analysis of *xyn5* from the genome-sequenced reference strain *T. reesei* QM6a shows that it encodes a non-functional, truncated form of XYN5. However, non-truncated orthologues are present in other genome sequenced *Trichoderma* spp., and sequencing of *xyn5* in other *T. reesei* wild-type isolates shows that they harbor a putative functional *xyn5* allele. In silico analysis and 3D modeling revealed that the encoded XYN5 has significant structural similarities to xylanases of the GH11 family, including a GH-typical substrate binding groove and a carboxylate pair in the active site. The *xyn5* of wild-type strain TUCIM1282 was recombinantly expressed in a *T. reesei* strain with a (hemi)cellulase-free background and the corresponding protein purified to apparent homogeneity. The pH and temperature optima and the kinetic parameters of the purified XYN5 were pH 4, 50 °C, and *V*_max_ = 2646 nkat/mg with a *K*_m_ of 9.68 mg/ml. This functional *xyn5* allele was used to replace the mutated version which led to an overall increase of the xylanolytic activity. These findings are of particular importance as GH11 xylanases are of high biotechnological relevance, and *T. reesei* is one of the main industrial producers of such lignocellulose-degrading enzymes.

**Electronic supplementary material:**

The online version of this article (doi:10.1007/s00253-017-8161-4) contains supplementary material, which is available to authorized users.

## Introduction

Due to its wide biotechnological application, the cellulose- and hemicellulose-degrading enzyme system of the filamentous fungus *Trichoderma reesei* has attracted many researchers and was consequently studied in considerable detail. Enzyme yields can exceed 100 g/l in industrial *T. reesei* fermentations, but the production and formulation of enzyme mixes still remains a bottleneck in the commercialization of products derived from lignocellulosic biomass. It is a peculiarity that virtual all strains used in industry and academia are derived from a single isolate *T. reesei* QM6a, which is regarded as the *T. reesei* reference strain and for which the genome sequence is available (Cherry and Fidantsef 2003; Martinez et al. 2008; Gupta et al. 2014; Bischof et al. 2016). Besides its potent cellulolytic enzyme machinery, *T. reesei* produces several xylanases which efficiently degrade xylan, the major component of hemicellulose and the second most abundant renewable biomaterial in plant biomass (Saha 2003; Ramoni and Seiboth 2016). In total, six xylanases (XYN1–6) are encoded in its genome belonging to the glycoside hydrolase (GH) families 10, 11, and 30 following the carbohydrate-active enzyme (CAZyme) classification (Lombard et al. 2014). The description of the first two GH11 family xylanases XYN1 and XYN2 dates back more than two decades (Tenkanen et al. 1992; Törrönen et al. 1992). These GH11 members are called “true” xylanases because they exclusively catalyze endo-β-1,4 cleavage (EC 3.2.1.8) of xylan. Enzymes in this family are particularly advantageous for various biotechnological applications, as they are small in size (∼20 kDa) and have high catalytic efficiencies with varying temperature and pH optima (Paës et al. 2012). XYN3 belongs to the GH10 family (Nakazawa et al. 2016; Wang et al. 2014), which exhibits endo-β-1,4-xylanase activity, but can also show endo-β-1,3-xylanase (EC 3.2.1.32) or xyloglucan/xyloglucosyl transferase activity (EC 2.4.1.207) as observed for plants (Derba-Maceluch et al. 2015). The GH30 XYN4 exhibits not only clear exo-activity but also some endo-activity, releasing mainly d-xylose from various linear xylooligosaccharides and xylans from the reducing end (Tenkanen et al. 2013). The appendage-dependent glucuronoxylan hydrolase XYN6, also of GH30 family, shows high specific activity on xylans and xylooligosaccharides that contain d-glucuronic acid or 4-*O*-methyl-d-glucuronic acid side substituents in an almost identical manner as bacterial GH30 xylanases do (Biely et al. 2014). However, in contrast to its bacterial counterparts, fungal XYN6 also revealed low activity on unsubstituted xylan and acidic xylooligosaccharides.

Transcription of the different xylanase genes depends beside other transcription factors mainly on the presence of the cellulase and xylanase regulator xylanase regulator 1 (XYR1; Stricker et al. 2006; Herold et al. 2013) and their induction is found in the presence of different sugars: Transcriptional regulation of the two GH11 family members revealed that *xyn1* is induced by different monomeric breakdown products of xylan including d-xylose and l-arabinose. *Xyn2* is constitutively expressed and can be further induced by d-xylose, in addition to common cellulase inducers such as cellulose and sophorose (Herold et al. 2013; Mach et al. 1996; Zeilinger et al. 1996). Similar to *xyn1*, *xyn4* is induced by d-xylose and l-arabinose (Herold et al. 2013). Potential conflicting results to former studies regarding inducing carbon sources can be explained by the observation that these xylanases are easily repressed in a carbon catabolite repressor 1 (CRE1)-dependent manner by higher concentrations of the two pentoses and that they are only inducible in the presence of lower concentrations (Herold et al. 2013). *Xyn3* and *xyn6* are exclusively induced by typical cellulase inducers such as cellulose, l-sorbose, or sophorose but not by xylan-derived carbon sources (Herold et al. 2013; Xu et al. 2000; Jonas Ramoni and Bernhard Seiboth unpublished data).

A further GH11 xylanase, *xyn5*, was overlooked during the *T. reesei* QM6a genome annotation (Martinez et al. 2008) and only later reported in a transcriptome analysis of conidiation regulated genes (Metz et al. 2011). Although its transcript was detected in a number of studies (Bischof et al. 2013; Häkkinen et al. 2012; Herold et al. 2013; Ivanova et al. 2013), the corresponding enzyme was not found in the *T. reesei* secretome (Herpoël-Gimbert et al. 2008; Peciulyte et al. 2014; Saloheimo and Pakula 2012), purified or biochemically characterized. Our analysis of the *xyn5* sequence and its corresponding protein of the genome sequenced *T. reesei* strain QM6a provides an explanation for this. In silico data shows that only a truncated, non-functional protein is encoded by this gene. However, sequencing of several *xyn5* alleles from other *T. reesei* wild-type strains revealed that these harbor a putative functional version of this xylanase in their genomes. Here we report the recombinant expression and biochemical characterization of a full-length XYN5 in a *T. reesei* strain with a (hemi)cellulase-free background (Uzbas et al. 2012). The produced XYN5 was purified to apparent homogeneity and its substrate specificities and enzymatic profile determined. In addition, replacement of the mutated version of *xyn5* by a functional *xyn5* allele led to a significant improvement of the overall xylanolytic activity.

## Materials and methods

### Strains and growth conditions

Strains used were the wild-type strain *T. reesei* QM6a (ATCC 13631) and different recombinant strains derived from the early cellulase mutant QM9414 (ATCC 26921) including Δ*xyr1* (Stricker et al. 2006) and Δ*tku70* which were constructed as described (Guangtao et al. 2009), with the only difference that the selection marker for *tku70* deletion was *ptrA* (Kubodera et al. 2002). QM9414 is derived from QM6a and was obtained by two rounds of irradiation in a linear particle accelerator (Mandels et al. 1971).

Other *T. reesei* wild-type strains were TUCIM283 (listed also under: C.P.K.283 or G.J.S.97-178), TUCIM665 (C.P.K.665 or TUB F-733), TUCIM936 (C.P.K.936 or G.J.S.89-7), and TUCIM1282 (C.P.K.1282, G.J.S.85-249 or CBS142139) from the TU Collection of Industrially Important Microorganisms and were described previously (Druzhinina et al. 2010; Kuhls et al. 1996; Samuels et al. 2012). Strain propagation, selection of transformants, and purification were performed on potato dextrose agar (PDA, Difco, Detroit, MI, USA) using 100 μg/ml hygromycin B (Carl Roth GmbH + Co. KG, Karlsruhe, Germany) as selective agent when needed. For liquid culture experiments, strains were grown in 250 ml Mandels-Andreotti (MA) medium (Mandels and Andreotti 1978) adjusted to pH 5 and supplemented with 1% (*w*/*v*) carbon source in 1-l Erlenmeyer flasks on a rotary shaker at 28 °C and 250 rpm. *Escherichia coli* Stellar™ (Clontech Laboratories, Inc., Mountain View, CA, USA) cells were used for plasmid construction and amplification.

### Sequencing of *T. reesei xyn5* alleles

Fungal DNA was extracted as previously described (Liu et al. 2000; Seiboth et al. 2004). *Xyn5* including 1.3 kb promoter and 0.7 kb terminator region was amplified by PCR from genomic DNA with oligonucleotides Infuse_xyn5_for and Infuse_xyn5_rev (Supplementary Table S1) using Phusion High-Fidelity DNA Polymerase (Thermo Scientific, Waltham, MA, USA). Sequencing of the *xyn5* coding region was performed by Sanger DNA sequencing at Microsynth AG (Balgach, Switzerland).

### Protein analysis

In silico analysis of the encoded proteins was performed using SignalP 4.1 Server (http://www.cbs.dtu.dk/services/SignalP/), NetNGlyc 1.0 Server (http://www.cbs.dtu.dk/services/NetNGlyc/), and NetOGlyc 4.0 Server (http://www.cbs.dtu.dk/services/NetOGlyc/). The KEX2 processed aa sequence was used to predict the protein structure using I-TASSER (Roy et al. 2010; Yang et al. 2015; Zhang 2008) and PyMOL (The PyMOL Molecular Graphics System, Version 1.3r1 Schrödinger, LLC. 2010).

### Transcriptional analysis

Strains were grown for 24 h on MA medium containing 1% (*v*/*v*) glycerol as carbon source. Subsequently, mycelia were collected, washed, and transferred to MA medium without carbon source. Following 30 min of incubation, d-xylose or l-arabinose to a final concentration of 1 mM was added to these cultures. Samples of mycelia were taken directly before adding the carbon source and 2, 4, and 6 h after addition. As control, strains were also cultured without carbon source addition. Mycelia were harvested, frozen in liquid nitrogen, and stored at −80 °C. Following RNA extraction (Chomczynski and Sacchi 1989), 5 μg of total RNA was reversely transcribed to complementary DNA (cDNA) using the RevertAid H minus first-strand cDNA synthesis kit (Thermo Scientific) with a 1:1 mixture of the supplied oligo-dT and random hexamer primers. The RT-qPCR reaction was performed in triplicates in an Eppendorf RealPlex^2^ Mastercycler (Eppendorf, Hamburg, Germany) in 96-well plates as described (Herold et al. 2013) using the transcription elongation factor 1α gene *tef1* for normalization. Relative gene expression was analyzed using REST software (Pfaffl et al. 2002). Oligonucleotides are found in Supplementary Table S1. Results are from at least two biological replicates with technical triplicates.

### Fungal strain construction

For expression of *xyn5* from *T. reesei* TUCIM1282, its coding region and terminator region was amplified from genomic DNA using primers infuse_*xyn5*_for and infuse_*xyn5*_rev. The 1.4 kb amplicon was introduced by recombinational cloning using the InFusion® HD Cloning Kit (Clontech Laboratories, Inc.) in the *Cla*I digested pLH_*hph*1_cDNA1 (Uzbas et al. 2012). The resulting plasmid p_*xyn5*oe contains the *hphB* expression cassette for selection of transformants on hygromycin B and *xyn5* under the control of the *T. reesei cDNA1* promoter region which is highly active in glucose grown cultures (Nakari-Setälä and Penttilä 1995). Ten micrograms of circular p_*xyn5*oe was transformed in *T. reesei* Δ*xyr1* using protoplast transformation (Gruber et al. 1990). Transformants were selected on PDA plates containing 100 μg/ml hygromycin B and purified by single spore isolation on plates containing 0.1% (*v*/*v*) Triton X-100. Genomic DNA from purified strains was isolated (Liu et al. 2000), and integration of the *xyn5* overexpression cassette was verified by PCR using primers seq_*xyn5*_3_for and seq_cDNA1_vec_rev. For positive strains amplification of a 1.2-kb fragment comprising the *cDNA1* promoter region, the *xyn5* open reading frame, and terminator was observed (Supplementary Fig. S1a).

The non-functional *xyn5* present in the QM6a strain line was replaced in *T. reesei* Δ*tku70* by the TUCIM1282 *xyn5* allele. Therefore, a 2.7-kb fragment containing 1.3 kb of the upstream and 0.7 kb of the downstream region of *xyn5* was amplified from genomic DNA of TUCIM1282 using oligonucleotides p1f_p/t*xyn5*_tgpd and P2R_T*xyn5*. This amplicon was introduced by InFusion® HD cloning into the *Xho*I digested pLH_*hph* (Hartl et al. 2007) which contains a hygromycin B resistance cassette for selection. The resulting vector was designated p*xyn5*hr. Using oligonucleotides P3F_Xyn5_5′flank and P4R_Xyn5_5′flank, 1.1 kb of a further upstream region of *xyn5* was amplified and cloned into the *Xma*I digested p*xyn5*hr resulting in p_*xyn5*hr_flank. Primers Pop_Xyn5F and Pop_Xyn5R were used to amplify the 6.2 kb *xyn5* replacement cassette which was transformed into swelling conidia of *T. reesei* Δ*tku70* by electroporation (Schuster et al. 2012). Transformants were selected and purified as described above. For verification of the integration of the full-length *xyn5* of TUCIM1282 at the endogenous *xyn5* locus in *T. reesei* Δ*tku70*, PCR was applied using oligonucleotides PF_*xyn5*_tricho_col and PR_*xyn5*_tricho_col. These primers bind in the 5′ flanking region of *xyn5* spanning the introduced *hphB* cassette. The amplicon of positive transformants was therefore 3 kb long, whereas the amplicon of the wild-type locus was 0.6 kb long. In case of an ectopic integration, both amplicons were detected (Supplementary Fig. S1b). Replacement was further verified by PCR amplification of *xyn5* and restriction analysis of the amplicon (Supplementary Fig. S1c, e). Estimation of the *xyn5* gene copy number (Supplementary Fig. S1d) was performed by qPCR (Tisch et al. 2011). The introduced *xyn5* coding region was finally verified by sequencing using p1f_p/t*xyn5*_tgpd and P2R_T*xyn5* for amplification and Ver_*xyn5*_1 for sequencing. The two individual positive transformants +*xyn5* A and +*xyn5* B were used in the further experiments. All oligonucleotides are found in Supplementary Table S1.

### XYN5 production and purification

Transformants harboring the *xyn5* expression cassette were grown in MA medium containing 1% d-glucose. Culture supernatants were harvested, filtered through Miracloth (Calbiochem, San Diego, CA, USA) and stored at 4 °C. The supernatant of these strains was analyzed for the presence of an around 19 kDa band representing XYN5 by SDS-PAGE (data not shown). For enzyme purification, culture supernatants of strain *xyn5g1* harvested after 36 h of cultivation were passed through a 0.22-μm pore size filter (Steritop Filter, Millipore, Billerica, MA) following concentration using Amicon Ultra filter units with 10 kDa cutoff membranes (Millipore, Billerica, MA, USA). XYN5 was then purified using a Mono S HR5/5 column (GE Bioscience, Chalfont, UK) which was equilibrated according to the operating manual with 10 mM NaAc pH 4.5 (buffer A). Bound proteins were eluted with a linear NaCl gradient ranging up to 0.1 M NaCl. Fractions were analyzed by xylanase activity tests and SDS-PAGE. Purified XYN5 protein concentration was determined by Nanodrop spectrophotometer (Thermo Scientific, Vienna, Austria). To analyze if XYN5 is glycosylated, the purified protein was treated with Endo-T (Stals et al. 2012) for 12 h at 28 °C. All culture supernatants were stored at 4 °C until further use.

### Xylanase assays

For the enzymatic characterization of XYN5 the enzyme was purified as described above. Xylanase activity was measured with the DNS method with 1.5% (*w*/*v*) beechwood xylan (Sigma-Aldrich, St. Louis, MO, USA; Product number X4252) dissolved in 10 mM NaAc buffer pH 4 (Bailey et al. 1992; König et al. 2002). For all reactions, the reaction volume was adjusted according to König et al. (2002) to fit 1.5-ml tubes. All reactions were done in triplicates, and d-xylose was used as standard to calculate the amount of released sugars. One unit was defined as the enzyme amount liberating 1 μmol of reducing sugars in 1 min under the given conditions. Non-linear fitting for determination of the enzyme activity was performed using SigmaPlot v13.0 (Systat Software Inc., San Jose, CA). For the determination of the optimal temperature, xylanase activity was determined in 10 mM NaAc buffer (pH 4) using different temperatures from 30 to 70 °C. For the determination of the pH optimum, McIlvaine buffer was used from pH 2.5 to 6.5 and reactions were performed at 50 °C.

To determine the enzymatic activity in the *xyn5* transformants +*xyn5*a and +*xyn5*b and the Δ*tku70* reference strain, strains were cultured on MA medium containing either 1% (*w*/*v*) lactose, beechwood xylan, or steam exploded wheat straw (kindly provided by Alexander Jäger of the University of Applied Sciences Upper Austria, FH Wels) in biological duplicates. Culture supernatants were filtered through Miracloth and stored at 4 °C until further use. Biomass was determined in technical triplicates by pelleting 1.5 ml of culture biomass which was subsequently washed with tap water, pelleted again, and dried to constant weight at 80 °C. For the determination of the endo-1,4-ß-d-xylanase activity, the chromogenic substrate S-AXBL (Megazyme International Ireland, Wicklow, Ireland) was used. Reactions were performed as described in the manufacturer’s manual but volumes were reduced 2.5 times to fit 1.5-ml reaction tubes.

### Mass spectrometry

Protein identification was carried out on an UltrafleXtreme (Bruker Daltonik, Bremen, Germany) after in-gel digestion, by MS and MS/MS analysis. In brief, after excising the gel lanes and removing the Coomassie staining by incubating the gel pieces in acetonitrile/100 mM NH_4_HCO_3_ (pH 8.5) (1/1, *v*/*v*), the samples were reduced with 10 mM dithiothreitol, alkylated with 50 mM iodoacetamide and trypsinized (20 ng trypsin, porcine, Roche, Basel, Switzerland). After overnight digestion, peptides were extracted with acetonitrile/water, dried in a vacuum centrifuge, and micropurified with C18 ZipTips (Merck Millipore, Billerica, MA, USA) and then eluted onto a stainless steel target together with α-cyano-4-hydroxy-cinnamic acid (CHCA, 3 mg/ml in 50% acetonitrile containing 0.1% trifluoroacetic acid). For all enzymatic digestion data, autolytic tryptic products, keratin and gel blank artifacts were assigned and removed before database search using an in-house Mascot server (Perkins et al. 1999). The database search was performed with the following parameters: taxonomy fungi, monoisotopic mass values, peptide mass tolerance of ±0.3 Da, two missed cleavages, carboxyamidomethylation as fixed modification, and methionine oxidations as variable modification. A protein was considered correctly identified if the search result was above the statistical threshold for the peptide mass fingerprint and all respective sequencing experiments.

### DNA sequences

*T. reesei xyn5* nucleotide sequence data are available in the DDBJ/EMBL/GenBank under the accession numbers KX139136 (TUCIM283), KX139137 (TUCIM665), KX139138 (TUCIM938), KX139139 (TUCIM1282/CBS 142139), KX455497 (QM6a), and KX455498 (RUT C-30).

## Results

### A non-functional version of *xyn5* is present in the reference strain *T. reesei* QM6a

**Fig. 1:**
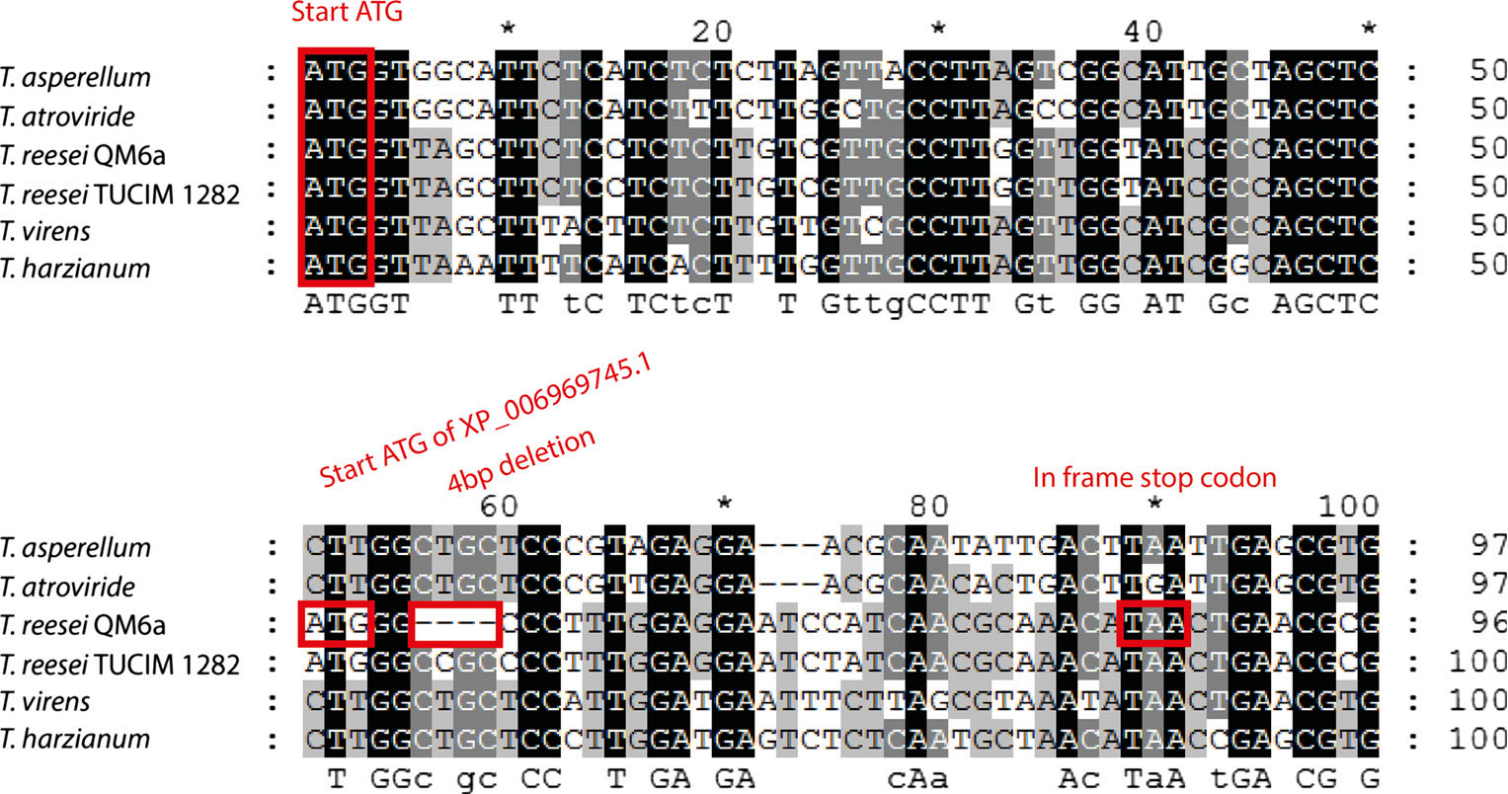
Alignment of the first 100 nucleotides of *xyn5* coding region of different *Trichoderma* spp. and *Trichoderma reesei* wild-type strains. Nucleotides were aligned and differences were manually annotated. When translated from the conserved start ATG found in all *xyn5* genes, the ORF of *T. reesei* QM6a misses 4 bp which leads to a frameshift and the termination of the ORF by a TAA stop codon. The start codon of the original NCBI database entry (XP_006969745.1) is found 46 bp downstream which misses therefore the XYN5 signal peptide sequence. GenBank accession numbers for *xyn5* are KX455497 (*T. reesei* QM6a), KX139139 (*T. reesei* TUCIM1282). The other sequences were retrieved from the JGI Genome Portal MycoCosm (http://genome.jgi.doe.gov/programs/fungi/index.jsf) and are Trias1|83211 (*Trichoderma asperellum*), Triat2|46014 (*Trichoderma atroviride*), TriviGv29_8_2|65505 (*Trichoderma virens*), and Triha1|118868 (*Trichoderma harzianum*). Residues highlighted by a *black background* are conserved in at least 90%, while residues highlighted by *gray* in 40% of the *xyn5* sequences

By microarray analysis and genome mining, a yet uncharacterized xylanase, designated as *xyn5*, was discovered in *T. reesei* (Metz et al. 2011). A sequence comparison of the encoded protein, deposited under GenBank ID XP_006969745.1, to the XYN5 orthologues of other genome-sequenced *Trichoderma* spp. revealed that the deposited protein sequence of the *T. reesei* reference strain QM6a is truncated. Following this annotation, a 152 aa protein is produced as the reading frame is interrupted by a stop codon which leads to a loss of the C-terminal part including one of the two conserved glutamates of the active site (Supplementary Fig. S2). However, this alignment also revealed that at the N-terminus of XYN5, a signal peptide for export in the endoplasmic reticulum is missing. According to our sequence analysis which is based on the comparison of the *T. reesei* QM6a *xyn5* to other orthologues, an ATG about 50 bp upstream from the annotated start ATG represents the true start codon (Fig. 1). But even when we used this start codon, we did not obtain a full-length XYN5 and the size of the encoded XYN5 was even reduced to 28 aa for *T. reesei* QM6a (Supplementary Fig. S2). This truncation of XYN5 is not the result of a sequencing error as our resequencing of the QM6a *xyn5* confirmed the deposited nucleotide sequence. Additional evidence comes from the *xyn5* sequence of the QM6a mutant RUT-C30 (GenBank ID: ETR96953.1) which is identical to the *xyn5* of strain QM6a. As sequence variations between different wild-type *T. reesei* strains can be considerably high (Linke et al. 2015), we sequenced the *xyn5* coding region of the four *T. reesei* wild-type strains TUCIM283, TUCIM665, TUCIM938, and TUCIM1282. Sequence analysis shows that in these strains, the *xyn5* orthologues translate into a 226 aa protein which is in accordance to the size of the XYN5 orthologues found in *Trichoderma atroviride* (225 aa) and *Trichoderma virens* (226 aa) (Supplementary Fig. S2). The encoded XYN5 proteins in *T. reesei* TUCIM938 and TUCIM1282 have a 100% aa identity while XYN5 from TUCIM283 shows a 99% (224 of 226) and XYN5 from TUCIM665 a 98% (222 of 226) aa sequence identity to the XYN5s of TUCIM1282 and TUCIM938. The aa sequence identity of the XYN5s of *T. reesei* TUCIM938 and TUCIM1282 to the XYN5s of *T. atroviride* and *T. virens* is only 87 and 90% respectively. Comparison of the TUCIM1282 and different *Trichoderma* spp. *xyn5* sequences to that of strain QM6a identified a number of nucleotide differences responsible for the truncation found in strain QM6a. These differences include a four nucleotide deletion in the 5′ part of the *xyn5* coding region leading to a premature translational stop resulting in the 28 aa XYN5 version of *T. reesei* QM6a (Fig. 1). In addition, a C ➔ T transition leads to the truncation in the NCBI deposited XYN5 of strain QM6a (Supplementary Fig. S3).

### Transcriptional regulation of *xyn5*

**Fig. 2:**
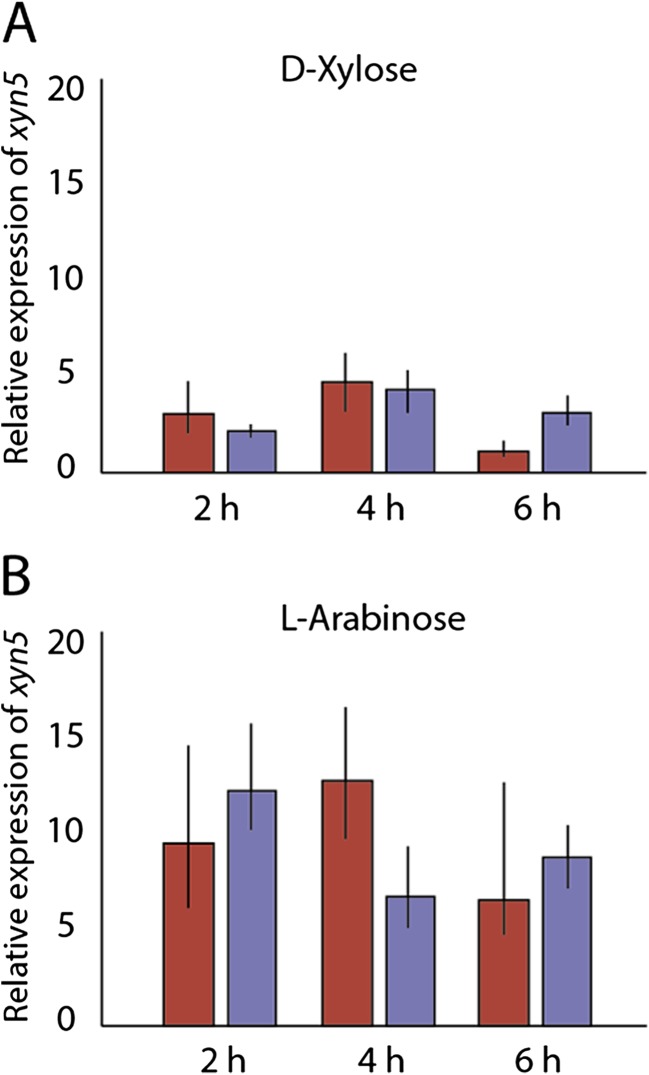
Transcriptional regulation of *xyn5* in *T. reesei* TUCIM1282 (*violet bars*) and QM6a (*red bars*). Strains were grown for 24 h on 1% (*v*/*v*) glycerol as carbon source and then transferred to medium with 1 mM *d*-xylose (**a**) and 1 mM *l* *-*arabinose (**b**). The fold change of *xyn5* expression in QM6a and TUCIM1282 was measured 2, 4 and 6 h after transfer to the inducing medium and normalized to the expression of *tef1*. *Xyn5* transcript levels were compared to the corresponding time points of cultures without a carbon source. Values represent the mean ± SD of at least two biological replicates (color figure online)

To address if *xyn5* is expressed in *T. reesei* TUCIM1282, we performed a comparative transcriptional analysis under xylanase inducing conditions using d-xylose and l-arabinose as potential inducers. This analysis showed that *xyn5* is inducible by both pentoses in TUCIM1282, whereby transcript levels on l-arabinose were higher compared to d-xylose (Fig. 2). We also tested *xyn5* transcription in *T. reesei* QM6a and found a comparable carbon source-dependent regulation which is in line with earlier results for *xyn5* transcription in the QM6a derivative QM9414 (Herold et al. 2013).

### In silico analysis and structural modeling of XYN5

**Fig. 3:**
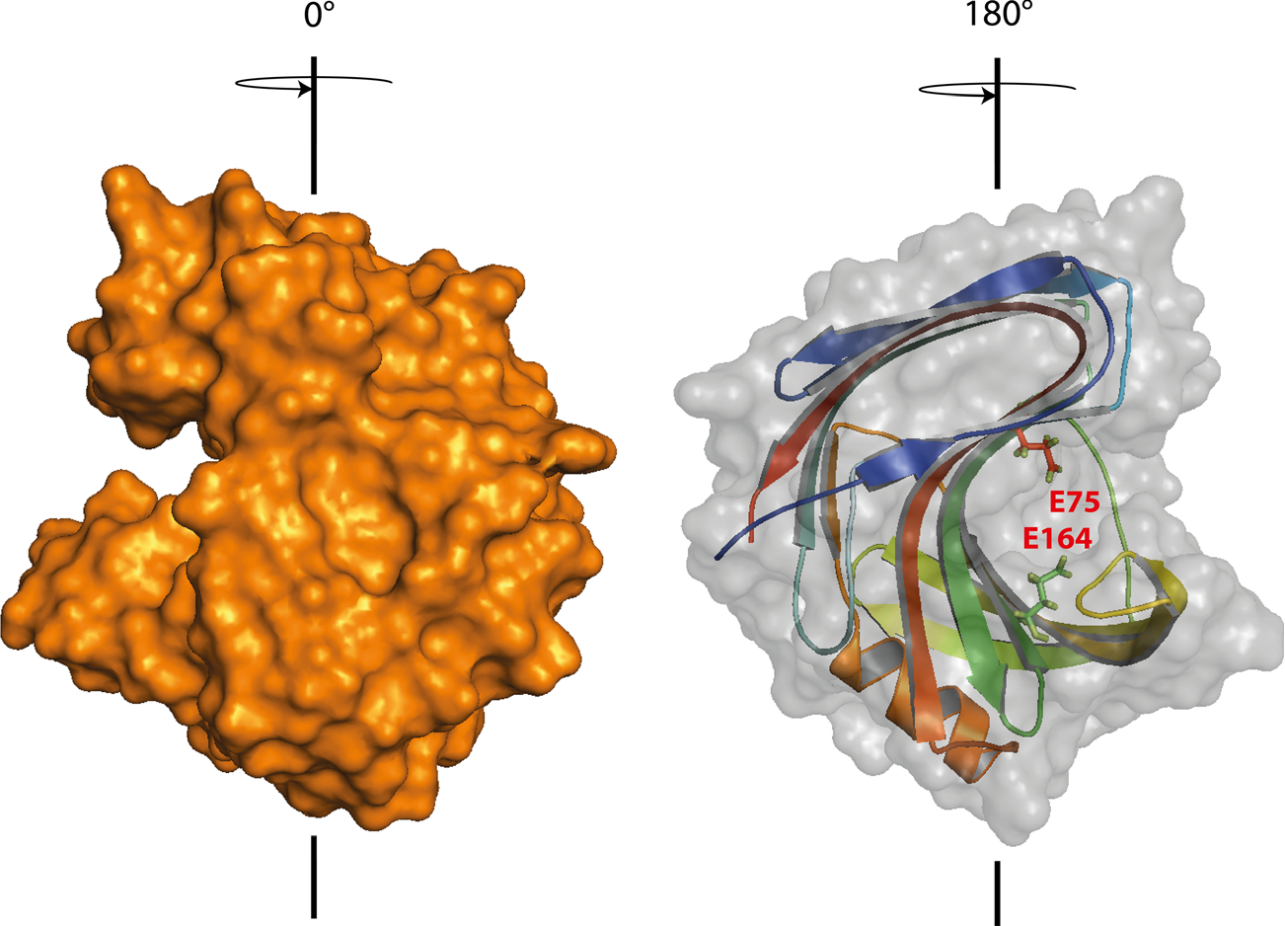
3D surface model of XYN5 from *T. reesei* TUCIM1282 based on the structure of XYN1 (PDB: 1XYN). The protein is primarily composed of GH11 typical antiparallel β-sheets with a unique α-helix. These secondary structures form a substrate binding cleft, which is illustrated on the *left side*. The two GH typical catalytic residues, *E75* and *E164*, are located within this cleft (*right side*)

According to previous reports which used the truncated XYN5 in their analysis (Häkkinen et al. 2012), it belongs to the GH11 family. The full-length protein consists of 226 aa including a 19 aa signal sequence leading to a protein of a predicted molecular mass of 24 kDa. The deduced protein shows higher sequence identity to XYN1 (69% aa identity) than to XYN2 (48% aa identity) of *T. reesei*. A comparison with the homologous *T. reesei* XYN1 revealed that XYN5 contains a putative dibasic (R-R) cleavage site for the endopeptidase KEX2 at position 47 and 48 (Supplementary Fig. S2). The KEX2 processed aa sequence was then used to predict the protein structure using I-TASSER and PyMOL. The analysis revealed typical GH11 structural motifs: the protein is formed mainly by antiparallel β-sheets with one α-helix. The β-sheets are thereby packed against each other and form the substrate binding cleft where the active site with two glutamic acids (E75 and E164) is present (Fig. 3) (Biely et al. 1997; Törrönen and Rouvinen 1997). Furthermore, three potential N-glycosylation sites (position 29, 75, 97) but no O-glycosylation site are found. In accordance with the other *T. reesei* GH11 xylanases, XYN5 lacks also a carbohydrate binding module.

### Recombinant expression and biochemical characterization of XYN5

**Fig. 4:**
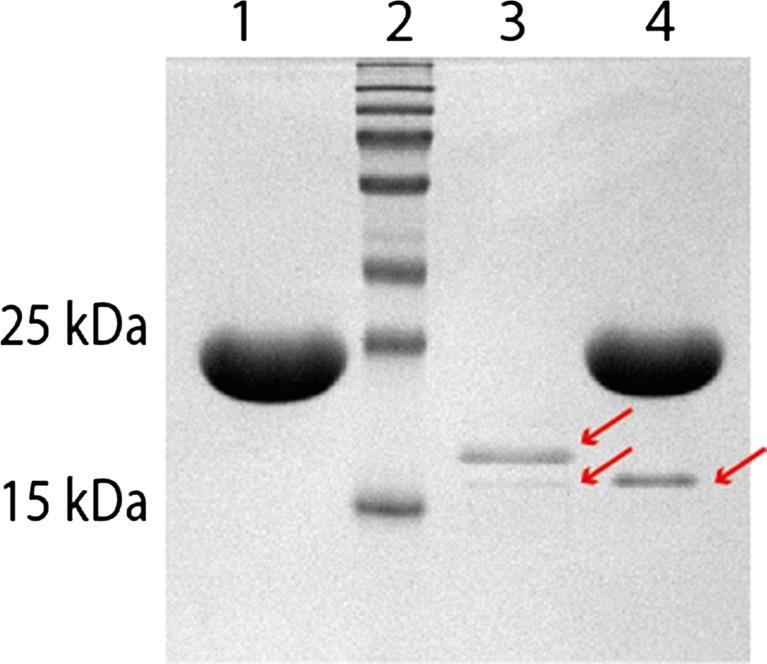
SDS-PAGE of the purified and deglycosylated XYN5 expressed in *T. reesei* strain *xyn5g1*. Following purification of XYN5 by cation exchange chromatography, two bands corresponding to *T. reesei* TUCIM1282 XYN5 were detected. This double band was reduced to a single band by Endo-T treatment of the XYN5 fraction. Lanes: *1*, Endo-T; *2*, molecular size marker; *3*, purified XYN5 protein (2 μg) after cation exchange chromatography; *4*, Endo-T treated purified XYN5 (2 μg)

**Fig. 5:**
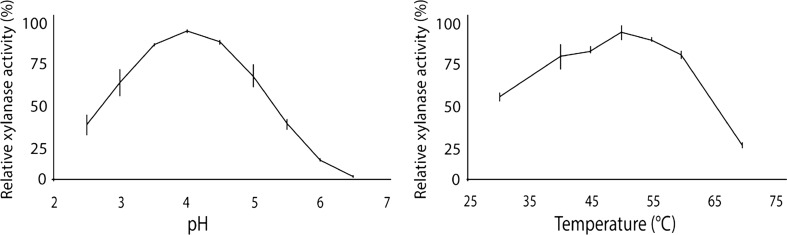
pH and temperature optimum of the recombinantly expressed XYN5 from *T. reesei* TUCIM1282 in *T. reesei* Δ*xyr1*. For determination of the pH optimum, beechwood xylan was dissolved in 0.1 M McIlvaine buffer and DNS-based assays were performed at 50 °C. For the determination of the temperature optimum, the substrate was dissolved in 10 mM NaAc buffer pH 4. Highest enzyme activity was detected at pH 4 and 50 °C. Mean ± SD of two biological replicates are shown

To demonstrate that *xyn5* of *T. reesei* TUCIM1282 encodes a functional xylanase, *xyn5* was recombinantly overexpressed under the control of the *cDNA1* promoter region in *T. reesei* strain Δ*xyr1* (Uzbas et al. 2012) to guarantee a xylanase free environment caused by the deletion of the major xylanase regulator *xyr1*. Purified *T. reesei* transformants were analyzed for the integration of the *xyn5* expression cassette (Supplementary Fig. S1a) and the production of an additional protein of about 19 kDa in the supernatant compared to the expression host Δ*xyr1*. The supernatant of the positive strain *xyn5*g1 was chosen for purification of XYN5. The small number of background proteins present in the supernatant of strain *xyn5*g1 could be removed by cation exchange chromatography. Following this procedure, two protein bands found around 21 and 19 kDa were detected by SDS-PAGE (Fig. 4). Analysis of the two protein bands by MS/MS identified both as XYN5 (Supplementary Fig. S4). Following the treatment of the respective XYN5 fractions with Endo-T, an endo-*N*-acetyl-β-d-glucosaminidase hydrolyzing the linkage between the two *N-*acetylglucosamine units of N-glycans (Stals et al. 2012), the larger XYN5 band disappeared, indicating that the double band is the result of a differentially N-glycosylated XYN5 (Fig. 4). The purified fractions of XYN5 were then pooled to determine the enzymatic characteristics. The optimal pH of 4 for XYN5 (Fig. 5) was similar to the reported pH optima for XYN1 between 3.5 and 4.5 (Table 1) and thereby lower than XYN2 with optima between 4.5 and 5.5. Highest XYN5 activity was measured at 50 °C (Fig. 5), whereas for XYN1 40 °C and for XYN2 45 °C were reported. The *V*_max_ of purified XYN5 at pH 4 and 50 °C on beechwood xylan was 2646 nkat/mg (Supplementary Fig. S5).

**Table 1:** Comparison of XYN5 to the other GH11 xylanases of *T. reesei*

Xylanase	*M*_r_	aa identity to XYN5	pH optimum	Temp. opt. (°C)	*K*_m_ (mg/ml)	*V*_max_ (nkat/mg)	Reference
XYN1	19 19	69%	3.5–4.0 4.0–4.5	n.a. 40	0.22 14.8–22.3	1667 1191–4736	Törrönen et al. (1992) Tenkanen et al. (1992)
XYN2	21 20	48%	4.5–5.5 5.0–5.5	n.a. 45	0.14 3–6.8	26,672 12,784–40,680	Törrönen et al. (1992) Tenkanen et al. (1992)
XYN5	18	100%	4.0	50	9.68 ± 1.26	2646 ± 142	This study

*n.a.* not available

### Reintroduction of a functional *xyn5* allele in *T. reesei* QM9414 increases total xylanase activity

**Fig. 6:**
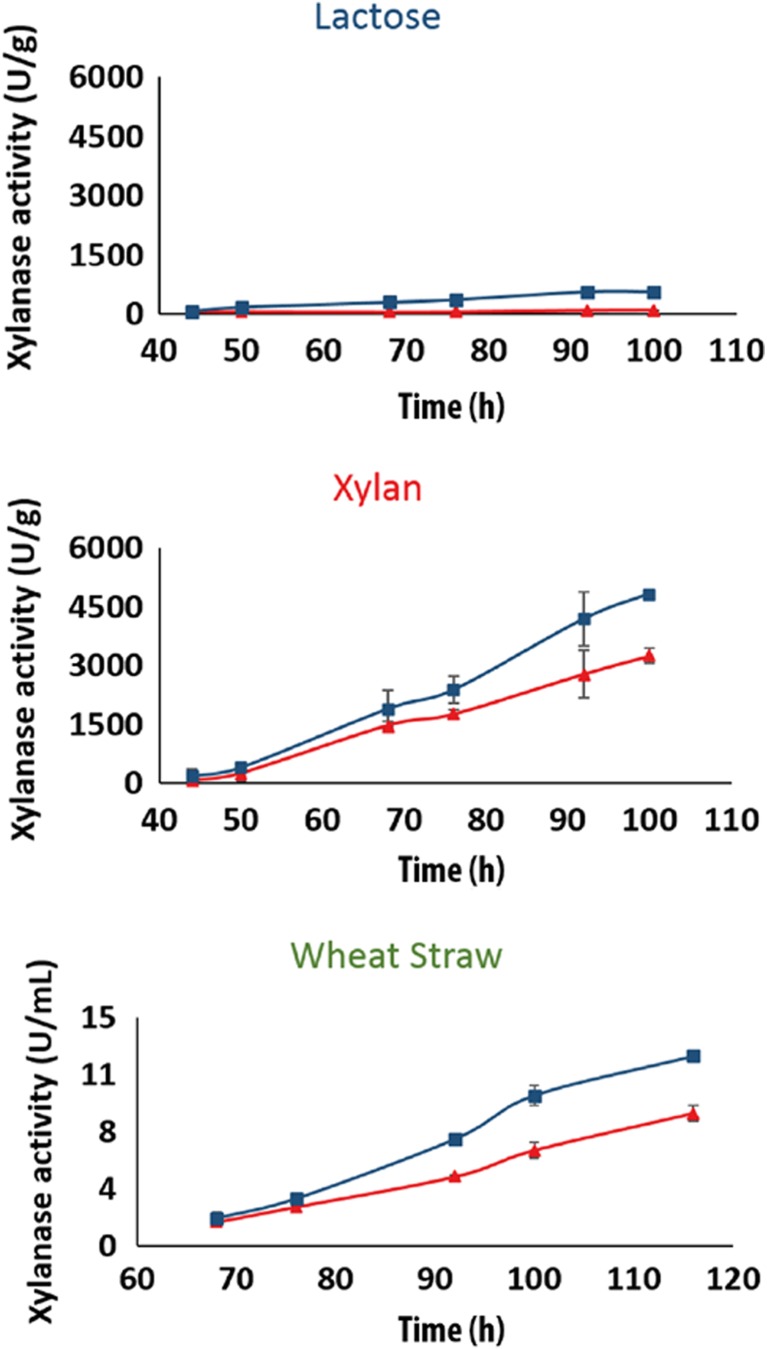
Increase in xylanolytic activity upon homologous replacement of the non-functional *xyn5* in *T. reesei* strain Δ*tku70* by the *T. reesei* TUCIM1282 *xyn5*. Total xylanase activity of the strains harboring the full-length *xyn5* (*blue line*) was compared to the parental strain Δ*tku70* (*red line*) during growth on MA medium using beechwood xylan, lactose, or wheat straw as carbon source. Lactose and beechwood xylan samples represent the activities corrected for biomass, whereas wheat straw samples represent volumetric activities. Mean ± SD of two biological replicates is shown (color figure online)

The finding that *xyn5* of *T. reesei* TUCIM1282 encodes a functional xylanase prompted us to analyze the effect of a reintroduction of the *xyn5* allele into the *T. reesei* QM6a strain line. Therefore, we used as recipient strain a Δ*tku70* strain of the early cellulase mutant QM9414 (Mandels et al. 1971) and homologously exchanged its *xyn5* with the *xyn5* allele of strain TUCIM1282 under control of its native promoter region. Positive transformants were analyzed for homologous recombination at the *xyn5* locus and for single copy integration of the *xyn5* allele (Supplementary Fig. S1b–e). The recombinant strains were then analyzed for xylanase activity on different xylanase inducing substrates including beechwood xylan, wheat straw, and the soluble carbon source lactose. On all substrates, the overall endo-xylanase activity of the recombinant strains was improved compared to the parental strain (Fig. 6). Highest xylanase activities were recorded on beechwood xylan, where the reintroduction of full-length *xyn5* improved the overall xylanolytic activity by 58% in comparison to the parental strain. The improvements in the overall xylanolytic activity were particularly present at later time-points of cultivation. This is in line with the finding that *xyn5*, together with *xyn1*, are highly expressed at low pH values which are typically reached at later stages of cultivation (Häkkinen et al. 2015).

## Discussion

In the *T. reesei* genome, three xylanases belonging to the GH11 family are encoded. Two of them were already characterized in the 1990s (Tenkanen et al. 1992; Törrönen et al. 1992), while the third GH11 xylanase XYN5 was discovered only recently. Obviously, due to an improper annotation as a glycoside hydrolase family 18 protein, it was missing from the original publication of the genome sequence of the *T. reesei* reference strain QM6a (Martinez et al. 2008). Only later during the manual annotation of transcripts expressed during conidiation, this third xylanase of the GH11 family was discovered. Although transcription of *xyn5* was reported, the enzyme did not appear in any proteomic or biochemical study (Borin et al. 2015; Dos Santos Castro et al. 2014; Jun et al. 2013; Peciulyte et al. 2014; Saloheimo and Pakula 2012). Based on our comparative analysis of *xyn5* alleles and their corresponding aa sequences from different *Trichoderma* spp. and *T. reesei* wild-type strains, we show that the deposited protein sequence of the reference strain QM6a is too short since a start codon further upstream of the originally proposed start codon encodes the most likely beginning of the protein sequence. We also identified several nucleotide differences within the coding region of *xyn5* from *T. reesei* QM6a which result in a very small truncated protein. However, the *xyn5* alleles of four other *T. reesei* strains encode a full-length protein which indicates that the relevant mutations leading to the truncated form of XYN5 happened specifically and recently in *T. reesei* strain QM6a history.

This full-length XYN5 aa sequence reveals a higher structural similarity to the already described XYN1 compared to XYN2. But all three GH11 xylanases are highly similar in size and exhibit comparable biochemical properties. A direct comparison of the kinetic properties is difficult as the data found in different publications on XYN1 and XYN2 are quite divergent (Table 1). One possible explanation for this discrepancy might be the high variations observed for endo-1,4-β-xylanase assays when performed in different laboratories under non standardized conditions (Bailey et al. 1992). An interesting property of XYN5 is its temperature optimum. With 50 °C, it exceeds the temperature optimum of the other two GH11 xylanases and this enzyme seems therefore to be more suitable for reactions which run at higher temperatures.

Regarding the expression profile of *xyn5* in *T. reesei*, we show that its transcription responds to the presence of l-arabinose and d-xylose in the wild-type strains TUCIM1282 and QM6a similar to an earlier study in the early cellulase mutant *T. reesei* QM9414 (Herold et al. 2013). *Xyn5* is also upregulated during sporulation (Metz et al. 2011) and belongs to a group of xylanases which are induced by a broader spectrum of inducers. These include also typical cellulase inducers such as sophorose (Ghassemi et al. 2015). This is in contrast to the regulation of *xyn3* and *xyn6* which are exclusively induced by cellulases inducers (Herold et al. 2013; Xu et al. 2000).

Our study is a further example that a comparative approach within a species can be useful to identify mutated genes which have lost their function during evolution of the strain. In a previous study, we have identified mutations in the MAP kinase scaffold encoding *ham5* in *T. reesei* QM6a which resulted in a female sterile phenotype of this strain. This non-functional HAM5 prevented sexual reproduction within the industrial and academic strain lines whereas the reintroduction of *ham5* led to fertile strains (Linke et al. 2015). Here the reintroduction of *xyn5* led to an improvement of the overall xylanase activity. Both studies display good examples that comparative analysis of the genes and genomes of different *T. reesei* strains is able to reveal non-functional genes and thereby expand the potential biotechnological use of this fungus. This dormant potential of less characterized wild-type strains might be further applied to identify additional non-functional CAZymes to improve the hydrolytic potential of this strain as this is still a major barrier towards cost-efficient production of second generation bioethanol. In addition, it can also be used to identify genes responsible for novel traits in the wild-type strains which are absent in the reference strain line of *T. reesei* QM6a. Such genes could be used, e.g., as dominant nutritional markers for genetic strain transformation. Introduction of these genes in the organism is described as cisgenesis, and the results are similar to conventionally strain breeding as genes are only transferred between closely related organisms. These homologous genes are considered to be advantageous over genes from heterologous origin, as expression of them usually does not need further optimization. They are preferred particularly in the food and feed industry over markers from other organisms or antibiotic markers as they facilitate the admission procedure for such products.

## Electronic supplementary material

ESM 1 (PDF 2437 kb)

## References

[CR1] Bailey M, Biely P, Poutanen K (1992). Interlaboratory testing of methods for assay of xylanase activity. J Biotechnol.

[CR2] Biely P, Vršanská M, Tenkanen M, Kluepfel D (1997). Endo-β-1,4-xylanase families: differences in catalytic properties. J Biotechnol.

[CR3] Biely P, Puchart V, Stringer MA, Mørkeberg Krogh KB (2014). *Trichoderma reesei* XYN VI—a novel appendage-dependent eukaryotic glucuronoxylan hydrolase. FEBS J.

[CR4] Bischof RH, Fourtis L, Limbeck A, Gamauf C, Seiboth B, Kubicek CP (2013). Comparative analysis of the *Trichoderma reesei* transcriptome during growth on the cellulase inducing substrates wheat straw and lactose. Biotechnol Biofuels.

[CR5] Bischof RH, Ramoni J, Seiboth B (2016). Cellulases and beyond: the first 70 years of the enzyme producer *Trichoderma reesei*. Microb Cell Factories.

[CR6] Borin GP, Sanchez CC, de Souza AP, de Santana ES, de Souza AT, Paes Leme AF, Squina FM, Buckeridge M, Goldman GH, Oliveira JV (2015). Comparative secretome analysis of *Trichoderma reesei* and *Aspergillus niger* during growth on sugarcane biomass. PLoS One.

[CR7] Cherry JR, Fidantsef AL (2003). Directed evolution of industrial enzymes: an update. Curr Opin Biotechnol.

[CR8] Chomczynski P, Sacchi N (1989). Single-step method of RNA isolation by acid guanidinium thiocyanate- phenol-chloroform extraction. Anal Biochem.

[CR9] Derba-Maceluch M, Awano T, Takahashi J, Lucenius J, Ratke C, Kontro I, Busse-Wicher M, Kosik O, Tanaka R, Winzéll A, Kallas Å, Leśniewska J, Berthold F, Immerzeel P, Teeri TT, Ezcurra I, Dupree P, Serimaa R, Mellerowicz EJ (2015). Suppression of xylan endotransglycosylase PtxtXyn10A affects cellulose microfibril angle in secondary wall in aspen wood. New Phytol.

[CR10] Dos Santos Castro L, Pedersoli WR, Antonieto AC, Steindorff AS, Silva-Rocha R, Martinez-Rossi NM, Rossi A, Brown NA, Goldman GH, Faca VM, Persinoti GF, Silva RN (2014). Comparative metabolism of cellulose, sophorose and glucose in *Trichoderma reesei* using high-throughput genomic and proteomic analyses. Biotechnol Biofuels.

[CR11] Druzhinina IS, Komon-Zelazowska M, Atanasova L, Seidl V, Kubicek CP (2010). Evolution and ecophysiology of the industrial producer *Hypocrea jecorina* (Anamorph *Trichoderma reesei*) and a new sympatric agamospecies related to it. PLoS One.

[CR12] Ghassemi S, Lichius A, Bidard F, Lemoine S, Rossignol MN, Herold S, Seidl-Seiboth V, Seiboth B, Espeso EA, Margeot A, Kubicek CP (2015). The ss-importin KAP8 (Pse1/Kap121) is required for nuclear import of the cellulase transcriptional regulator XYR1, asexual sporulation and stress resistance in *Trichoderma reesei*. Mol Microbiol.

[CR13] Gruber F, Visser J, Kubicek CP, De Graaff LH (1990). The development of a heterologous transformation system for the cellulolytic fungus *Trichoderma reesei* based on a *pyrG*-negative mutant strain. Curr Genet.

[CR14] Guangtao Z, Hartl L, Schuster A, Polak S, Schmoll M, Wang T, Seidl V, Seiboth B (2009). Gene targeting in a nonhomologous end joining deficient *Hypocrea jecorina*. J Biotechnol.

[CR15] Gupta VK, Schmoll M, Herrera-Estrella A, Upadhyay RS, Druzhinina I, Tuohy MG (2014). Biotechnology and biology of *Trichoderma*.

[CR16] Häkkinen M, Arvas M, Oja M, Aro N, Penttilä M, Saloheimo M, Pakula TM (2012). Re-annotation of the CAZy genes of *Trichoderma reesei* and transcription in the presence of lignocellulosic substrates. Microb Cell Factories.

[CR17] Häkkinen M, Sivasiddarthan D, Aro N, Saloheimo M, Pakula TM (2015). The effects of extracellular pH and of the transcriptional regulator PACI on the transcriptome of *Trichoderma reesei*. Microb Cell Factories.

[CR18] Hartl L, Kubicek CP, Seiboth B (2007). Induction of the *gal* pathway and cellulase genes involves no transcriptional inducer function of the galactokinase in *Hypocrea jecorina*. J Biol Chem.

[CR19] Herold S, Bischof R, Metz B, Seiboth B, Kubicek CP (2013). Xylanase gene transcription in *Trichoderma reesei* is triggered by different inducers representing different hemicellulosic pentose polymers. Eukaryot Cell.

[CR20] Herpoël-Gimbert I, Margeot A, Dolla A, Jan G, Mollé D, Lignon S, Mathis H, Sigoillot JC, Monot F, Asther M (2008) Comparative secretome analyses of two *Trichoderma reesei* RUT-C30 and CL847 hypersecretory strains. Biotechnol Biofuels 1(18). doi:10.1186/1754-6834-1-18 10.1186/1754-6834-1-18 PMC2631499 19105830

[CR21] Ivanova C, Baath JA, Seiboth B, Kubicek CP (2013). Systems analysis of lactose metabolism in *Trichoderma reesei* identifies a lactose permease that is essential for cellulase induction. PLoS One.

[CR22] Jun H, Guangye H, Daiwen C (2013). Insights into enzyme secretion by filamentous fungi: comparative proteome analysis of *Trichoderma reesei* grown on different carbon sources. J Proteome.

[CR23] König J, Grasser R, Pikor H, Vogel K (2002). Determination of xylanase, β-glucanase, and cellulase activity. Anal Bioanal Chem.

[CR24] Kubodera T, Yamashita N, Nishimura A (2002). Transformation of *Aspergillus* sp. and *Trichoderma reesei* using the pyrithiamine resistance gene (*ptrA*) of *Aspergillus oryzae*. Biosci Biotechnol Biochem.

[CR25] Kuhls K, Lieckfeldt E, Samuels GJ, Kovacs W, Meyer W, Petrini O, Gams W, Börner T, Kubicek CP (1996). Molecular evidence that the asexual industrial fungus *Trichoderma reesei* is a clonal derivative of the ascomycete *Hypocrea jecorina*. Proc Natl Acad Sci U S A.

[CR26] Linke R, Thallinger GG, Haarmann T, Eidner J, Schreiter M, Lorenz P, Seiboth B, Kubicek CP (2015). Restoration of female fertility in *Trichoderma reesei* QM6a provides the basis for inbreeding in this industrial cellulase producing fungus. Biotechnol Biofuels.

[CR27] Liu D, Coloe S, Baird R, Pedersen J (2000). Rapid mini-preparation of fungal DNA for PCR. J Clin Microbiol.

[CR28] Lombard V, Golaconda Ramulu H, Drula E, Coutinho PM, Henrissat B (2014). The carbohydrate-active enzymes database (CAZy) in 2013. Nucleic Acids Res.

[CR29] Mach RL, Strauss J, Zeilinger S, Schindler M, Kubicek CP (1996). Carbon catabolite repression of xylanase I (*xyn1*) gene expression in *Trichoderma reesei*. Mol Microbiol.

[CR30] Mandels M, Andreotti R (1978). The cellulose to cellulase fermentation. Proc Biochem.

[CR31] Mandels M, Weber J, Parizek R (1971). Enhanced cellulase production by a mutant of *Trichoderma viride*. Appl Microbiol.

[CR32] Martinez D, Berka RM, Henrissat B, Saloheimo M, Arvas M, Baker SE, Chapman J, Chertkov O, Coutinho PM, Cullen D, Danchin EGJ, Grigoriev IV, Harris P, Jackson M, Kubicek CP, Han CS, Ho I, Larrondo LF, De Leon AL, Magnuson JK, Merino S, Misra M, Nelson B, Putnam N, Robbertse B, Salamov AA, Schmoll M, Terry A, Thayer N, Westerholm-Parvinen A, Schoch CL, Yao J, Barbote R, Nelson MA, Detter C, Bruce D, Kuske CR, Xie G, Richardson P, Rokhsar DS, Lucas SM, Rubin EM, Dunn-Coleman N, Ward M, Brettin TS (2008). Genome sequencing and analysis of the biomass-degrading fungus *Trichoderma reesei* (syn. *Hypocrea jecorina*). Nat Biotech.

[CR33] Metz B, Seidl-Seiboth V, Haarmann T, Kopchinskiy A, Lorenz P, Seiboth B, Kubicek CP (2011). Expression of biomass-degrading enzymes is a major event during conidium development in *Trichoderma reesei*. Eukaryot Cell.

[CR34] Nakari-Setälä T, Penttilä M (1995). Production of *Trichoderma reesei* cellulases on glucose-containing media. Appl Environ Microbiol.

[CR35] Nakazawa H, Kawai T, Ida N, Shida Y, Shioya K, Kobayashi Y, Okada H, Tani S, J-i S, Kawaguchi T, Morikawa Y, Ogasawara W (2016). A high performance *Trichoderma reesei* strain that reveals the importance of xylanase III in cellulosic biomass conversion. Enzym Microb Technol.

[CR36] Paës G, Berrin JG, Beaugrand J (2012). GH11 xylanases: structure/function/properties relationships and applications. Biotechnol Adv.

[CR37] Peciulyte A, Anasontzis GE, Karlström K, Larsson PT, Olsson L (2014). Morphology and enzyme production of *Trichoderma reesei* Rut C-30 are affected by the physical and structural characteristics of cellulosic substrates. Fungal Genet Biol.

[CR38] Perkins DN, Pappin DJ, Creasy DM, Cottrell JS (1999) Probability-based protein identification by searching sequence databases using mass spectrometry data. Electrophoresis 20(18):3551–3567. doi:10.1002/(sici)1522-2683(19991201)20:18%3C3551::aid-elps3551%3E3.0.co;2-2 10.1002/(SICI)1522-2683(19991201)20:18<3551::AID-ELPS3551>3.0.CO;2-2 10612281

[CR39] Pfaffl MW, Horgan GW, Dempfle L (2002). Relative expression software tool (REST) for group-wise comparison and statistical analysis of relative expression results in real-time PCR. Nucleic Acids Res.

[CR40] Ramoni J, Seiboth B, Druzhinina IS, Kubicek CP (2016). Degradation of plant cell wall polymers by fungi. Environmental and microbial relationships, the Mycota.

[CR41] Roy A, Kucukural A, Zhang Y (2010). I-TASSER: a unified platform for automated protein structure and function prediction. Nat Protoc.

[CR42] Saha BC (2003). Hemicellulose bioconversion. J Ind Microbiol Biotechnol.

[CR43] Saloheimo M, Pakula TM (2012). The cargo and the transport system: secreted proteins and protein secretion in *Trichoderma reesei* (*Hypocrea jecorina*). Microbiology.

[CR44] Samuels GJ, Ismaiel A, Mulaw TB, Szakacs G, Druzhinina IS, Kubicek CP, Jaklitsch WM (2012). The Longibrachiatum Clade of *Trichoderma*: a revision with new species. Fungal Divers.

[CR45] Schuster A, Bruno KS, Collett JR, Baker SE, Seiboth B, Kubicek CP, Schmoll M (2012) A versatile toolkit for high throughput functional genomics with *Trichoderma reesei*. Biotechnol Biofuels 5(1). doi:10.1186/1754-6834-5-1 10.1186/1754-6834-5-1 PMC3260098 22212435

[CR46] Seiboth B, Hartl L, Pail M, Fekete E, Karaffa L, Kubicek C (2004). The galactokinase of *Hypocrea jecorina* is essential for cellulase induction by lactose but dispensable for growth on D-galactose. Mol Microbiol.

[CR47] Stals I, Karkehabadi S, Kim S, Ward M, Van Landschoot A, Devreese B, Sandgren M (2012). High resolution crystal structure of the endo-N-acetyl-β-D-glucosaminidase responsible for the deglycosylation of *Hypocrea jecorina* cellulases. PLoS One.

[CR48] Stricker AR, Grosstessner-Hain K, Würleitner E, Mach RL (2006). Xyr1 (xylanase regulator 1) regulates both the hydrolytic enzyme system and D-xylose metabolism in *Hypocrea jecorina*. Eukaryot Cell.

[CR49] Tenkanen M, Puls J, Poutanen K (1992) Two major xylanases of *Trichoderma reesei*. Enzym Microb Technol 14. doi:10.1016/0141-0229(92)90128-B

[CR50] Tenkanen M, Vršanská M, Siika-Aho M, Wong DW, Puchart V, Penttilä M, Saloheimo M, Biely P (2013). Xylanase XYN IV from *Trichoderma reesei* showing exo- and endo-xylanase activity. FEBS J.

[CR51] Tisch D, Kubicek CP, Schmoll M (2011). The phosducin-like protein PhLP1 impacts regulation of glycoside hydrolases and light response in *Trichoderma reesei*. BMC Genomics.

[CR52] Törrönen A, Rouvinen J (1997). Structural and functional properties of low molecular weight endo-1,4-β-xylanases. J Biotechnol.

[CR53] Törrönen A, Mach RL, Messner R, Gonzalez R, Kalkkinen N, Harkki A, Kubicek CP (1992). The two major xylanases from *Trichoderma reesei*: characterization of both enzymes and genes. Nat Biotechnol.

[CR54] Uzbas F, Sezerman U, Hartl L, Kubicek CP, Seiboth B (2012). A homologous production system for *Trichoderma reesei* secreted proteins in a cellulase-free background. Appl Microbiol Biotechnol.

[CR55] Wang J, Zeng D, Mai G, Liu G, Yu S (2014). Homologous constitutive expression of Xyn III in *Trichoderma reesei* QM9414 and its characterization. Folia Microbiol (Praha).

[CR56] Xu J, Nogawa M, Okada H, Morikawa Y (2000). Regulation of *xyn3* gene expression in *Trichoderma reesei* PC-3-7. Appl Microbiol Biotechnol.

[CR57] Yang J, Yan R, Roy A, Xu D, Poisson J, Zhang Y (2015). The I-TASSER suite: protein structure and function prediction. Nat Methods.

[CR58] Zeilinger S, Mach RL, Schindler M, Herzog P, Kubicek CP (1996). Different inducibility of expression of the two xylanase genes *xyn1* and *xyn2* in *Trichoderma reesei*. J Biol Chem.

[CR59] Zhang Y (2008). I-TASSER server for protein 3D structure prediction. BMC Bioinformatics.

