# Supplementary material for: Trichoderma reesei xylanase 5 is defective in the reference strain QM6a but functional alleles are present in other wild-type strains
Source: Appl Microbiol Biotechnol. 2017 Feb 22;101(10):4139–49. doi: 10.1007/s00253-017-8161-4 (PMC5403845; doi:10.1007/s00253-017-8161-4)
Supplement: Supplementary file 1 — (PDF 2437 kb) [file 253_2017_8161_MOESM1_ESM.pdf]

Supplementary Material

Journal name: Applied Microbiology and Biotechnology

Manuscript title: *Trichoderma reesei* xylanase 5 is defective in the reference strain QM6a but functional alleles are present in other wild-type strains.

Author names: Jonas Ramoni[1], Martina Marchetti-Deschmann[2], Verena Seidl-Seiboth[1], Bernhard Seiboth[1,*]

Author Affiliations:

[1]Molecular Biotechnology, Research Division Biochemical Technology, Institute of Chemical Engineering, TU Wien, A-1060 Vienna, Austria.

[2]Institute of Chemical Technologies and Analytics, TU Wien, A-1060, Vienna, Austria

Corresponding author: Bernhard Seiboth, Research Division Biochemical Technology, Institute of Chemical Engineering, TU Wien, Gumpendorferstraße 1a, A-1060 Vienna, Austria.

Tel.: +43-1-58801-166553; email: bernhard.seiboth@tuwien.ac.at; website: http://www.vt.tuwien.ac.at/

Table S1. Oligonucleotides used in this study. Overhangs for InFusion® recombinational cloning are indicated in bold.

Name	Sequence	Reference
Infuse_*xyn5*_for	**CAACTTCTCTCATC**GATATGGTTAGCTTCTCCTCTC	This study
Infuse_*xyn5*_rev	**TGCAGGTCGACATCGAT**CACCAGCCTGTTCTTCACCG	This study
Seq_*xyn5*_3_for	TGCGTAGGAGACCATTTGCTTC	This study
Seq_cDNA1_vec_rev	GTTGTGTGGAATTGTGAGCGG	This study
P1f_p/t*xyn5*_tgpd	**CCATAGTACCCTCGAG**CGGATATCACCCTACTGTCC	This study
P2R_T*xyn5*	**CATTCCTTCTCTCGAG**TTCGTCACCAGCCTGTTC	This study
P3F_Xyn5_5'flank	**GAGCTCGGTACCCGGG**CCTCCCTATTACTTGTGTTTC	This study
P4R_Xyn5_5'flank	**CTCTCGGATCCCCGGGG**CACTTTGACCGGCAAG	This study
Pop_Xyn5F	GTTTTCCCAGTCACGACGTT	This study
Pop_Xyn5R	TTCTTGGATTTGCAGCACAG	This study
PF_xyn5_tricho_col	CAACTCACGAGACTTTCACTCG	This study
PR_xyn5_tricho_col	CTTGACCGGATGACGACTTT	This study
Ver_xyn5_1	CTTGGGCTTCTCTTTCAACG	This study
qPCR_XYN5_for2	CTACGGCTGGAGCACTAACC	Herold et al. (2013)
qPCR_XYN5_rev2	CTGGTGACGCTGCCCTTC	Herold et al. (2013)
qPCR_tef1_for	CCACATTGCCTGCAAGTTCGC	Herold et al. (2013)
qPCR_tef1_rev	GTCGGTGAAAGCCTCAACGCA	Herold et al. (2013)

Fig. S1

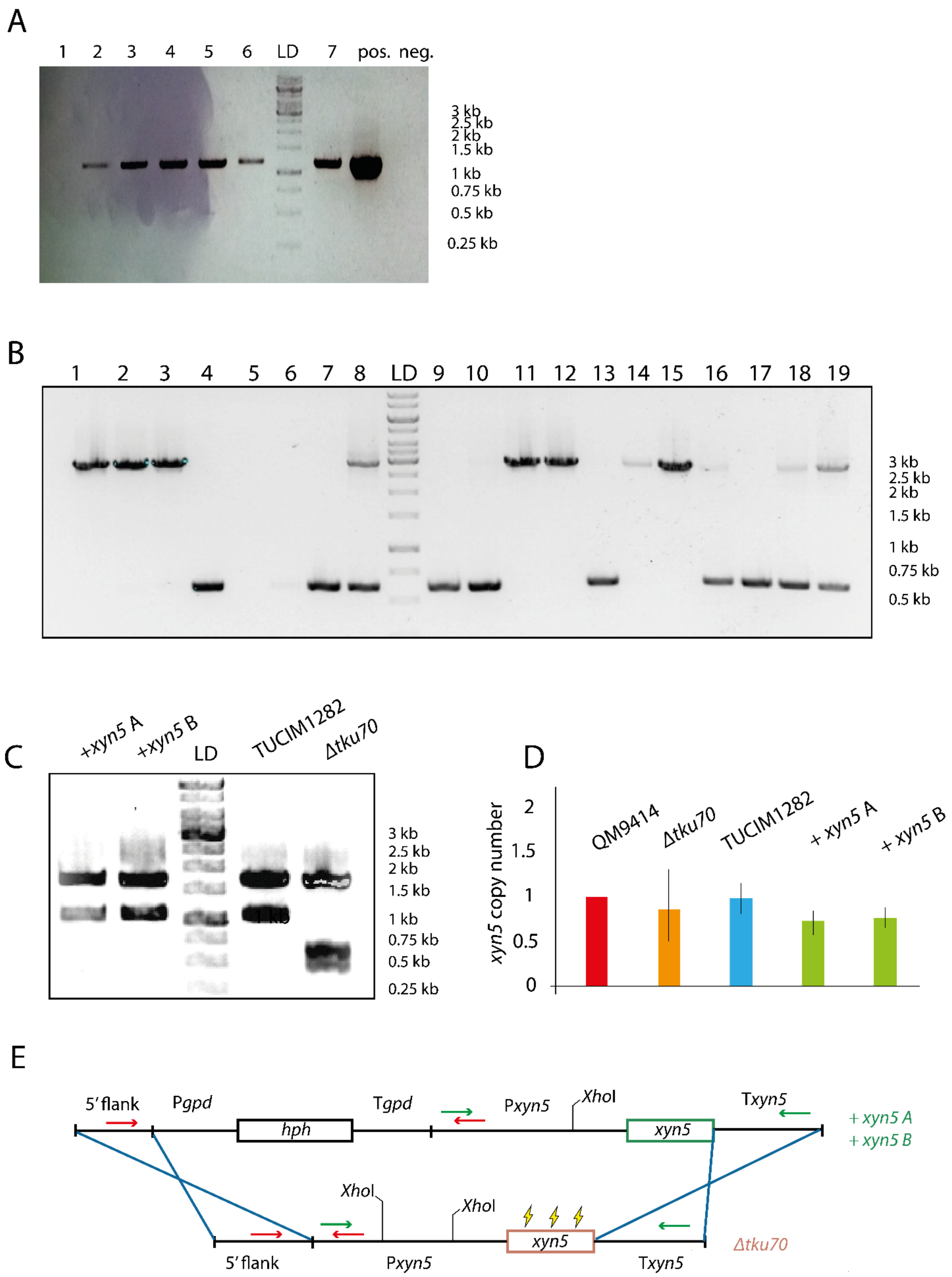
A
1 2 3 4 5 6 LD 7 pos. neg.
3 kb
2.5 kb
2 kb
1.5 kb
1 kb
0.75 kb
0.5 kb
0.25 kb
B
1 2 3 4 5 6 7 8 LD 9 10 11 12 13 14 15 16 17 18 19
3 kb
2.5 kb
2 kb
1.5 kb
1 kb
0.75 kb
0.5 kb
C
+xyn5 A
+xyn5 B
LD
TUCIM1282
Δtku70
3 kb
2.5 kb
2 kb
1.5 kb
1 kb
0.75 kb
0.5 kb
0.25 kb
D
xyn5 copy number
2
1.5
1
0.5
0
QM9414
Δtku70
TUCIM1282
+ xyn5 A
+ xyn5 B
E
5′flank
Pgpd
hph
Tgpd
Pxyn5
XhoI
xyn5
Txyn5
+ xyn5 A
+ xyn5 B
XhoI
XhoI
xyn5
5′flank
Pxyn5
Txyn5
Δtku70

Fig. S1 Analysis of *T. reesei* strains transformed with *xyn5* of TUCIM1282. (A) Genotyping PCR of *Δxyr1* strains transformed with p_xyn5oe for expression of the *T. reesei* TUCIM1282 *xyn5* under the *cDNA1* promoter. Presence of the *cDNA1-xyn5* expression cassette was tested with the primers seq_xyn5_3_for and seq_cdna1_vec_rev and resulted in the amplification of a 1.2 kb fragment in positive transformants. As positive control (pos.) p_xyn5oe was used and as negative control genomic DNA of strain *Δxyr1*. (B) Replacement of *xyn5* in *T. reesei Δtku70* by the functional *xyn5* allele of TUCIM1282. For verification of the integration of the full-length *xyn5* of TUCIM1282 at the endogenous *xyn5* locus oligonucleotides PF_*xyn5*_tricho_col and PR_*xyn5*_tricho_col were used which bind in the 5' flanking region of *xyn5* spanning the introduced *hphB* cassette. The amplicon of positive transformants is therefore 3 kb, whereas the amplicon of the endogenous locus of strain Δ*tku70* is 0.6 kb. Ectopic integration of the replacement cassette leads to the amplification of both amplicons. (C) Replacement of the endogenous *xyn5* was further verified by PCR amplification of *xyn5* and restriction analysis of the amplicon. The *xyn5* locus was thereby amplified with the primers PF_X5_cont and PR_X5_cont from the *xyn5* complemented Δ*tku70* strains +xyn5 A, +xyn5 B and the reference strains TUCIM 1282 and Δ*tku70*. The amplicons were digested with *Xho*I. The *xyn5* amplicon of the *Δtku70* strain contains two XhoI sites which lead to three bands of 0.6, 0.5 and 1.7 kb. In TUCIM 1282 and +xyn5 A and +xyn5 B only one *Xho*I site is found resulting in two bands of 1.1 and 1.7 kb. (D) *Xyn5* copy number determination in *T. reesei* +xyn5 A and +xyn5 B. To verify that *xyn5* of *T. reesei* TUCIM 1282 is present only as a single copy, the *xyn5* copy number of the +xyn5 A and +xyn5 B strains and the control strains QM9414, Δ*tku70* and TUCIM 1282 was determined by quantitative PCR. The intensities were normalized to an internal control (*cbh2*) as published before (Bischof et al. 2015). *T. reesei* strain QM9414 served as reference and the amplification intensity of *xyn5* was set to 1. (E) Schematic drawing of the *xyn5* replacement in *T. reesei* strain Δ*tku70*. Oligonucleotides used in this study for verification of the integration of *xyn5* of TUCIM 1282 are indicated. The red arrows represent the oligonucleotides PF_*xyn5*_tricho_col and PR_*xyn5*_tricho_col and the green arrows represent the oligonucleotides PF_X5_cont and PR_X5_cont.

Fig. S2

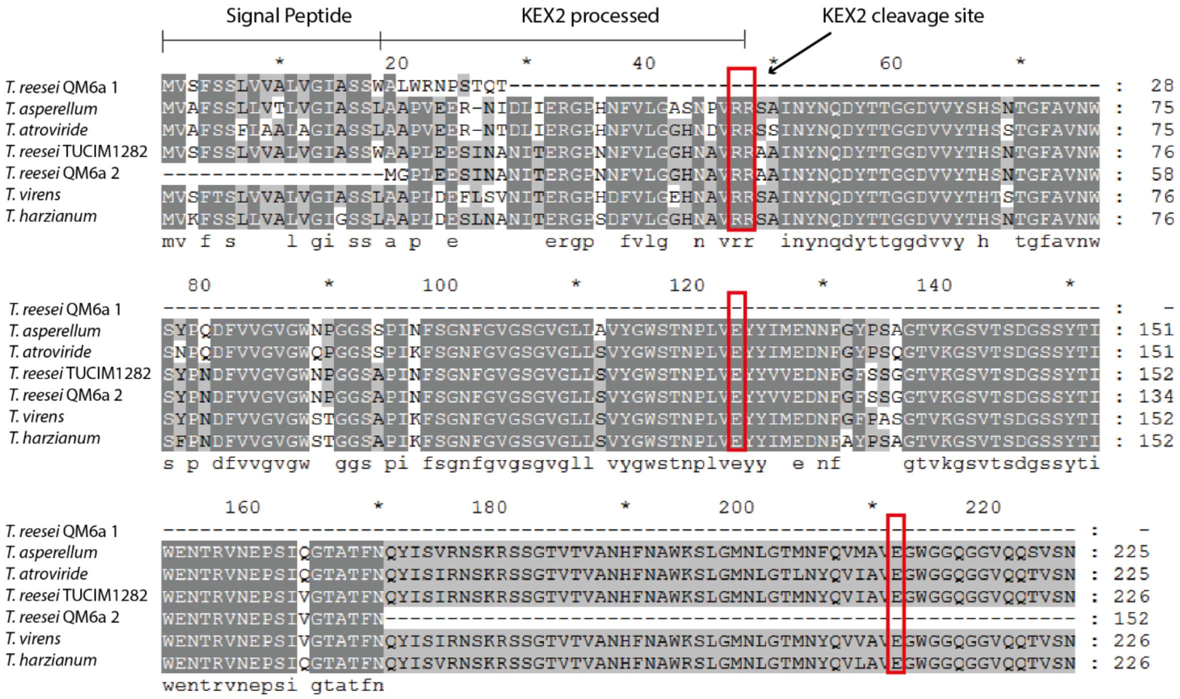

Fig. S2 Amino acid alignment of XYN5 from different *Trichoderma* spp. *and T. reesei* wild-type strains. Indicated are the N terminal signal peptide and the peptide which is released by the processing of the KEX2 protease which cleaves at the RR site highlighted in red. In addition, the two conserved glutamates (E) of the active site are highlighted. This reveals that according to the NCBI database entry the *T. reesei* QM6a XYN5 would miss the second glutamate because of an in-frame stop codon. For XYN5 of *T. reesei* QM6a the original NCBI version (EGR44310, marked by 2) and our manually edited version (ANW82584, marked by 1) are given. Accession numbers for XYN5 sequences are ID83211 (*T. asperellum*), XP_013945037.1 (*T. atroviride*), ANX99795 (*T. reesei* TUCIM1282), XP_013950942.1 (*T. virens*) and KKO99033.1 (*T. harzianum*).

Fig. S3

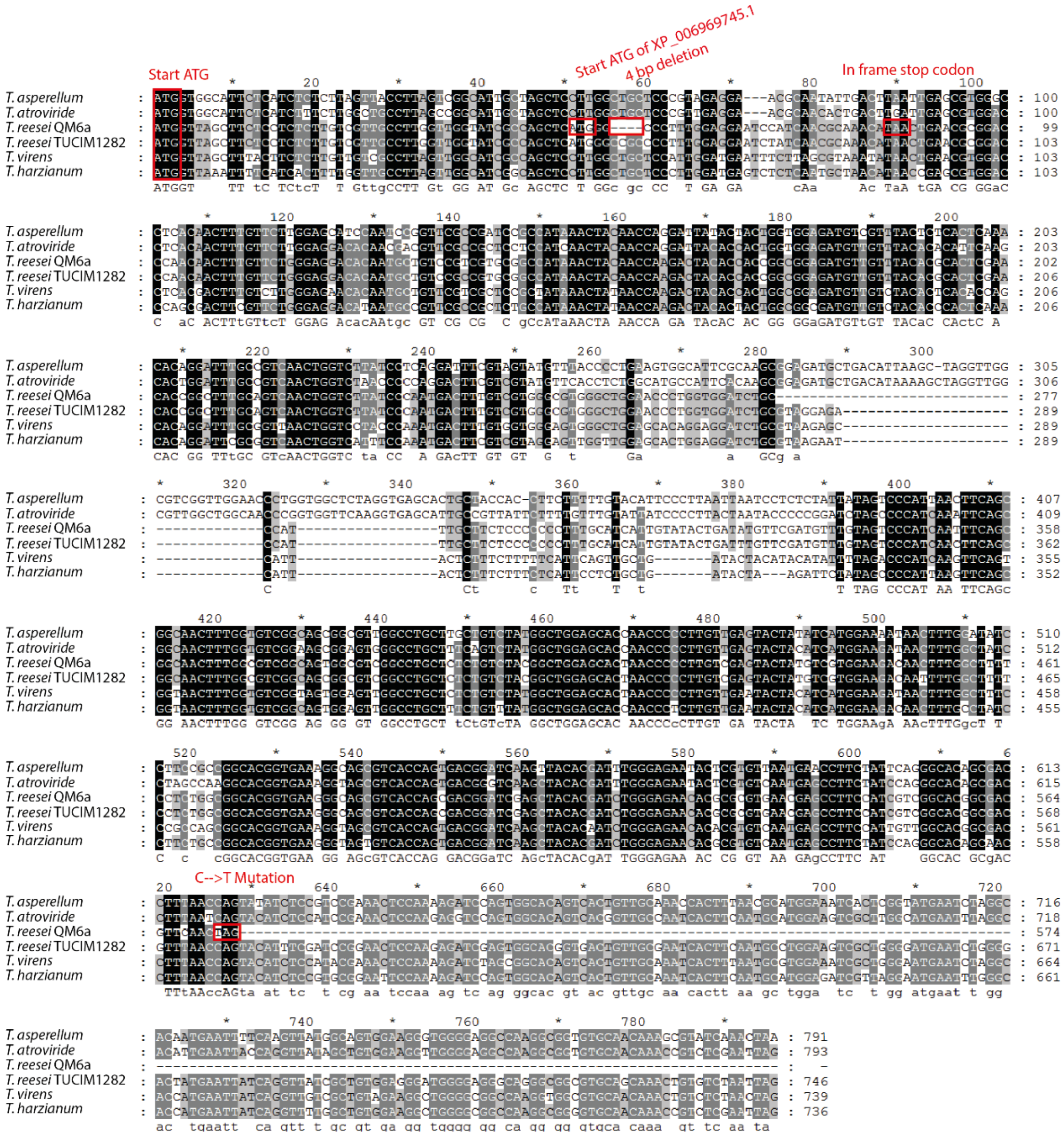

Fig. S3 Alignment of x*yn5* nucleotide sequences from different *Trichoderma* spp*. and T. reesei* wild-type strains. Nucleotides were aligned and differences were manually annotated. Indicated is a 4 bp deletion which leads to a frame-shift and the termination of the ORF by an in frame TAA stop codon. The start codon of the original NCBI database entry (XP_006969745) is found 46 bp downstream of the correct start codon. In addition, a C→T transition at position 572 leads to an in frame stop codon which leads to truncation of the C-terminal part of XYN5 in *T. reesei* QM6a.

Fig. S4

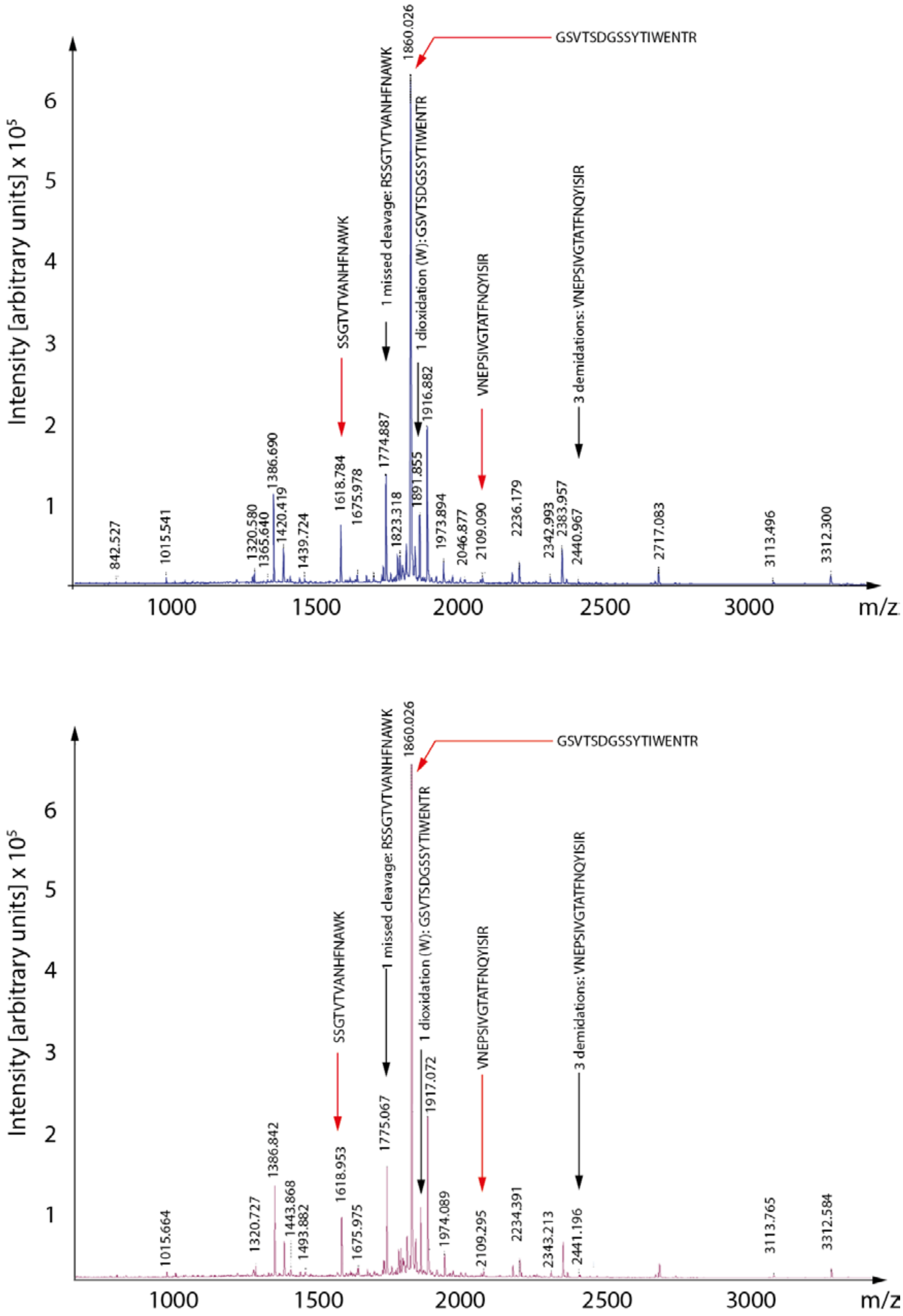

Intensity [arbitrary units] x 10^5
m/z
GSVTSDGSSYTIWENTR
1 missed cleavage: RSSGTVTVANHFNAWK
1 dioxidation (W): GSVTSDGSSYTIWENTR
SSGTVTVANHFNAWK
VNEPSIVGTATFNQYISIR
3 demidations: VNEPSIVGTATFNQYISIR
842.527
1015.541
1320.580
1365.640
1386.690
1420.419
1439.724
1618.784
1675.978
1774.887
1823.318
1860.026
1891.855
1916.882
1973.894
2046.877
2109.090
2236.179
2342.993
2383.957
2440.967
2717.083
3113.496
3312.300
GSVTSDGSSYTIWENTR
1 missed cleavage: RSSGTVTVANHFNAWK
1 dioxidation (W): GSVTSDGSSYTIWENTR
SSGTVTVANHFNAWK
VNEPSIVGTATFNQYISIR
3 demidations: VNEPSIVGTATFNQYISIR
1015.664
1320.727
1386.842
1443.868
1493.882
1618.953
1675.975
1775.067
1860.026
1917.072
1974.089
2109.295
2234.391
2343.213
2441.196
3113.765
3312.584

Predicted mass	Position in protein sequence	Trypsin digested peptide sequence
9680,53	1-92	AAINYNQDYTTGGDVVYTHSNTGFAVNWSYPNDFVVGVGWNPGGSAP INFSGNFGVGSGV GLLSVYGWSTNPLVEYYVVE DNFGFSSGGTVK
3195,51	148-178	SLGMNLGTMNYQVIAVEGWG GQGGVQQTVSN
2109,09	110-128	VNEPSIVGTATFNQYISIR
1859,84	93-109	GSVTSDGSSYTIWENTR
1618,79	133-147	SSGTVTVANHFNAWK

Fig. S4 Mass spectrometric analysis of XYN5. Following XYN5 purification two bands were detected in an SDS-PAGE (see Fig. 4). The two co-eluted proteins were excised from the SDS-PAGE gel and analyzed by MS/MS. Three of the five predicted peptides of an in silico trypsin digested XYN5 were detected in both protein bands and are highlighted in blue in the table.

Fig. S5

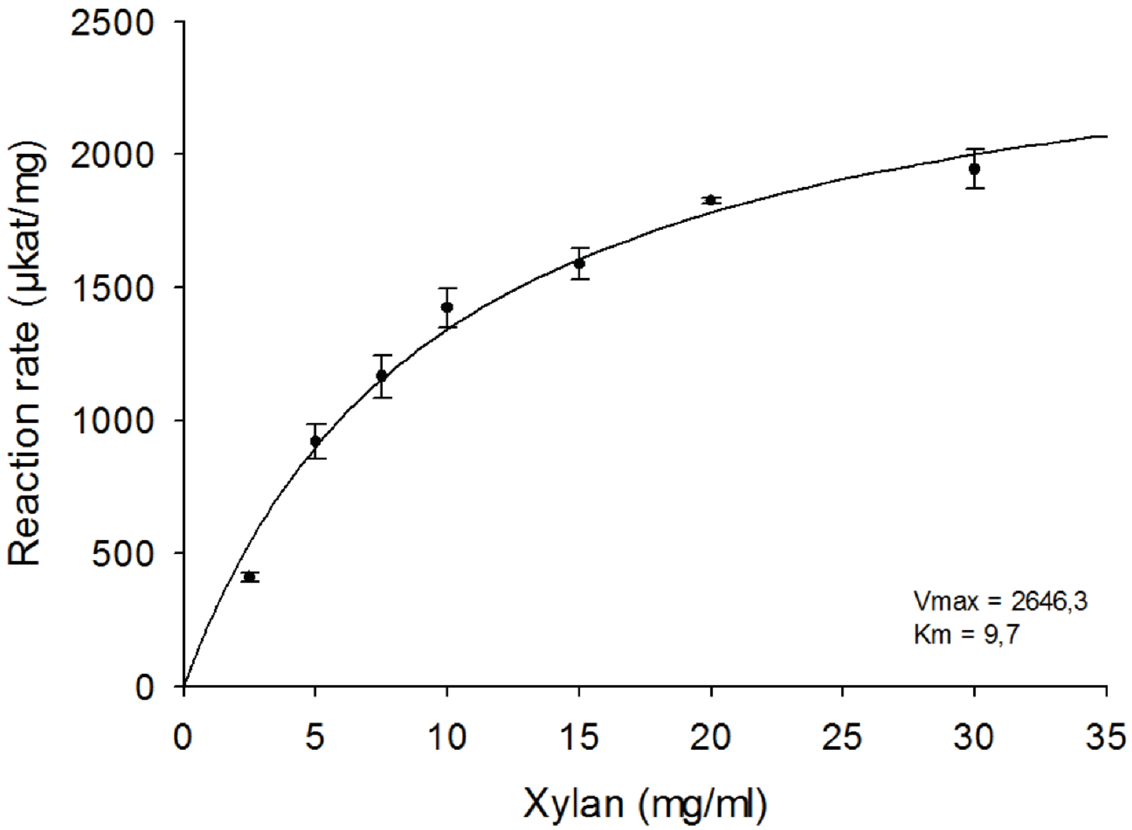

Fig. S5 Michaelis-Menten reaction kinetics of XYN5. The reaction kinetics (Km, v_{max}) were determined by elevating substrate concentrations up to 30 mg/ml beechwood xylan. The calculation of Km and v_{max} was performed using SigmaPlot 13.0

Reference:

Bischof RH, Horejs J, Metz B, Gamauf C, Kubicek CP, Seiboth B (2015) L-Methionine repressible promoters for tuneable gene expression in *Trichoderma reesei*. Microb Cell Fact 14(1):1-11. doi:10.1186/s12934-015-0308-3
